# A Noninvasive Tool to Assess the Distribution of Pacific Lamprey (*Entosphenus tridentatus*) in the Columbia River Basin

**DOI:** 10.1371/journal.pone.0169334

**Published:** 2017-01-09

**Authors:** Kellie J. Carim, J. Caleb Dysthe, Michael K. Young, Kevin S. McKelvey, Michael K. Schwartz

**Affiliations:** United States Department of Agriculture, Forest Service, National Genomics Center for Wildlife and Fish Conservation, Rocky Mountain Research Station, Missoula, Montana, United States of America; Bournemouth University, UNITED KINGDOM

## Abstract

The Pacific lamprey (*Entosphenus tridentatus*) is an anadromous fish once abundant throughout coastal basins of western North America that has suffered dramatic declines in the last century due primarily to human activities. Here, we describe the development of an environmental DNA (eDNA) assay to detect Pacific lamprey in the Columbia River basin. The eDNA assay successfully amplified tissue derived DNA of Pacific lamprey collected from 12 locations throughout the Columbia River basin. The assay amplifies DNA from other Entosphenus species found outside of the Columbia River basin, but is species-specific within this basin. As a result, the assay presented here may be useful for detecting *Entosphenus* spp. in geographic range beyond the Columbia River Basin. The assay did not amplify tissue or synthetically derived DNA of 14 commonly sympatric non-target species, including lampreys of the genus *Lampetra*, which are morphologically similar to Pacific lamprey in the freshwater larval stage.

## Introduction

The Pacific lamprey (*Entosphenus tridentatus*) is an anadromous fish that was once abundant along the Pacific Coast of North America from Alaska to Mexico [1]. However, human activities and infrastructure have reduced habitat and restricted fish passage leading to dramatic declines of Pacific lamprey populations throughout Washington, Oregon, Idaho, and California [2]. The Pacific lamprey, along with the western brook lamprey (*Lampetra richardsoni*) and western river lamprey (*L*. *ayresii*), was petitioned in 2003 for protection under the U. S. Endangered Species Act, but the petition was denied due to insufficient data on species distribution and population structure of these fish [3]. Techniques that can rapidly and reliably detect species at low densities and accurately distinguish between species (particularly in early life stages) are necessary to further inform the status of all lamprey in this context. To assist in this effort, we developed an eDNA assay for detecting Pacific lamprey in the Columbia River basin where state, tribal, and federal efforts for species recovery are underway.

## Methods and Results

To develop an eDNA assay specific to Pacific lamprey, we first sequenced DNA of the mitochondrial cytochrome *c* oxidase I (COI) of Pacific lamprey and unidentified freshwater lamprey. Tissue samples were obtained from previous studies conducted by the Columbia River Inter-Tribal Fish Commission, Nez Perce Tribe and the U.S. Forest Service. Freshwater lamprey species included in this study were obtained from various locations in the Willamette River basin, OR. DNA was extracted from tissue samples using the Qiagen DNEasy^®^ Blood and Tissue Kit following manufacturer’s protocol. We developed sequencing primers by aligning whole mitochondrial genome sequences of the least brook lamprey (*Lampetra aepyptera*), the American brook lamprey (*Lethenteron appendix*), and the European river lamprey (*Lampetra fluviatilis*; GenBank accession numbers: KP742974, KM267719, and Y18683 respectively) in MEGA 6 [4], and manually screening the COI region for conserved sequences among species. Within these conserved areas, we developed three sets of forward and reverse primers (Table 1), each amplifying a 534–600 base-segment of COI. PCR products for sequencing were obtained by amplifying DNA in 40.2 μl reaction volumes containing 1 mM of each primer, 2.5 mM MgCl_2_, 4 μl of 10X PCR buffer, 0.2 mM of each deoxynucleotide triphosphate (dNTP), 0.2 μl BSA, 0.2 μl 50X TITANIUM^™^ Taq DNA Polymerase (Clontech Laboratories), and the remaining volume with PCR-grade water. Cycling conditions consisted of 95°C/12 min, [95°C/1 min, 55°C/1 min, 72°C/1.5 min] × 35 cycles, with final extension at 72°C/5 min. PCR products were cleaned with Exo-SAP-IT (Affymetrix) and DNA sequence data were obtained using the Big Dye kit and the 3700 DNA Analyzer (ABI; High Throughput Genomics Unit, Seattle, WA). The sequence data were analyzed and concatenated into a 1 421 base fragment using Sequencher 5.3 (Gene Codes Corporation, Ann Arbor, MI USA http://www.genecodes.com) and were uploaded to GenBank (accession numbers: KX389871-KX389877; KX443679- KX443687).

**Table 1:** Primer sequences for amplification and sequencing of the COI region of the Pacific lamprey mitochondrial genome; and sequences for components of the eDNA assay.

Name	Laboratory application	Amplicon length	Sequence 5’- 3’
LAMP_COI_F1	Sequencing	600	GTGACTCTCATTCGTTGATTATTCTCTACTAA
LAMP_COI_R601			GTATAGTRATGGCGGCTGCAAGTAC
LAMP_COI_F523	Sequencing	549	ACTATAACACAATAYCAAACYCCTTTATTTGT
LAMP_COI_R1072			GAAGGATAATRTCTAGTGATGAGTTGGATAA
LAMP_COI_F976	Sequencing	534	ACTCTCCATGGCGGAAAAATC
LAMP_COI_R1510			TTGRACATARGCTGGTTCTTCATAAGT
EntTri_F	eDNA	126	TACCACTCATACTTAGTGCCCCTG
EntTri_R			CTGTGCCAGCCCCTGCT
EntTri_Probe			FAM-TTTGATTACTTCCACCCTCAC-MGBNFQ

*Lampetra* individuals were presumed to be either *L*. *ayresii* or *L*. *richardsoni* based on the location of capture. However, we were unable to identify these individuals to the species level due to the lack of resolution in the taxonomy of *Lampetra* across western North America [5].

In addition to the sequence data obtained above, we obtained sequences of the COI region from GenBank for three additional Pacific lamprey individuals as well as 19 non-target species that are closely related or commonly co-occur (Table 2). We used the *DECIPHER* package [6] in *R* v. 3.0.1 [7] to obtain primers specific to Pacific lamprey. We then aligned sequences in MEGA 6, and visually identified a species-specific region to create a hydrolysis probe with a MGB quencher (Table 1). Primers were manufactured by IDT and purified using standard de-salting methods. The probe was obtained from Life Technologies and was purified using HPLC. We assessed the melting temperatures of the primers (forward: 59.0°C; reverse: 59.5°C) and probe (70.0°C) in Primer Express 3.0.1 (Life Technologies) and screened for secondary structures using IDT OligoAnalyzer (https://www.idtdna.com/calc/analyzer).

**Table 2:** Species and source for DNA sequences used for *in silico* assay development. Source refers to GenBank accession number or location of collection for specimens analyzed in this study. Probe mismatches refers to the number of base-pair differences between the eDNA assay probe and the COI sequence for a particular species. Pacific brook lamprey (*Lampetra pacifica*) sequence data were obtained after primer and probe development.

Common name	Scientific name	Probe mismatches	Source
Pacific lamprey	*Entosphenus tridentatus*	0	GU440367; KF918874-KF918875; KX389871-KX389877
Mottled sculpin	*Cottus bairdii*	6	HQ557189; JN025020; JN025023; JN025026
Slimy sculpin	*Cottus cognatus*	6	JN025088; JN025095; JN025097; JN025099
Three-spined stickleback	*Gasterosteus aculeatus*	5	EU524634; HQ712384; KP823165; KR862768
Least brook lamprey	*Lampetra aepyptera*	2	JN026945
Western river lamprey	*Lampetra ayresii*	4	HQ010078
European river lamprey	*Lampetra fluviatilis*	1	KM286704; KM286706; KM286710
Kern brook lamprey	*Lampetra hubbsi*	4	HQ557301; JN025325-JN025327
Pacific brook lamprey	*Lampetra pacifica*	5	KY072805-KY72808
European brook lamprey	*Lampetra planeri*	1	KM286716; KM286719; KM373674; KM373681
Western brook lamprey	*Lampetra richardsoni*	4	JN026960-JN026961
Unidentified brook lamprey spp.	*Lampetra* spp.	4	KX443679- KX443687
Olympic mudminnow	*Novumbra hubbsi*	4	HQ557339; JN027849-JN027851
Cutthroat trout	*Oncorhynchus clarkii*	7	EU524198; EU524201; HQ557150; JN027854
Rainbow trout	*Oncorhynchus mykiss*	7	FJ999086; FJ999088; FJ999090; KM373668
Sockeye salmon	*Oncorhynchus nerka*	7	EU524223; EU524225; FJ999233; HQ712703
Chinook salmon	*Oncorhynchus tshawytcha*	6	EU524234; FJ164931; HQ712706; KF558293
Brown trout	*Salmo trutta*	8	KC501168; KM287114; KM287116; KM287119
Bull trout	*Salvelinus confluentus*	7	EU522399; EU522401; EU522403; EU524365
Brook trout	*Salvelinus fontinalis*	7	KM287121; KM287123; HQ960794; HQ961027
Dolly Varden	*Salvelinus malma*	7	EU522411; EU522413; EU522415; EU522417

We extracted DNA from tissue of 42 Pacific lamprey from 12 different locations in the Columbia River basin and 13 non-target species using methods outlined above (Table 3). DNA concentrations for tissues were measured using a Qubit 2.0 fluorometer (ThermoFisher Scientific) and ranged from 12.8–72 ng/μl. We were unable to obtain tissue from Pacific brook lamprey (*Lampetra pacifica*) for screening against the Pacific lamprey assay. To overcome this limitation, we used published sequence data (GenBank accessions KY072805-KY72808) to identify a DNA fragment matching a 449 basepair region of the COI gene encompassing the region of the Pacific lamprey assay. We then obtained a synthetic DNA fragment from IDT in the form of a plasmid with a PUVI restriction digest sequence inserted at the end of the 449 basepair sequence. The plasmid was linearized using PVUI restriction enzyme (New England Biosystems) following manufacturer’s protocol and purified using a PureLink^™^ PCR Micro Kit (Invitrogen). We estimated the concentration of the linearized plasmid DNA using a Qubit 2.0 fluorometer (ThermoFisher Scientific) and diluted the DNA to 0.04 ng/ul in TE. We screened each non-target tissue and the synthetic *L*. *pacifica* DNA against our assay in a single 15-μl reaction using a StepOne Plus Real-time PCR Instrument (Life Technologies). Each reaction contained 7.5 μl Environmental Master Mix 2.0 (Life Technologies), 900 nM forward primer, 900 nM reverse primer, 250 nM probe, 4 μl DNA template (diluted 1:100 from extract), and 2.75 μl deionized water. Thermocycler conditions included 95°C for 10 minutes followed by 45 cycles of denaturation at 95°C for 15s and annealing and extension at 60°C for 1 min. The assay amplified all Pacific lamprey samples (cycle threshold, C_t_, ranging from 19.1–21.9 depending on DNA concentration), and there was no amplification in 13 of the 14 non-target samples, including the synthetic *L*. *pacifica* DNA. One non-target species (Pit-Klamath brook lamprey, *E*. *lethophagus*) amplified (C_t_ = 21.7; DNA concentration = 11.9 ng/μl) with this assay. Publically available sequence data in the COI region for Klamath lamprey (*E*. *similis*; GenBank accessions JN025328-–JN025330, native to Klamath River basin in OR and CA) as well as Pit-Klamath brook lamprey (GenBank accessions HQ579097 and JN025328; native to Pit River drainage and Klamath River basin in OR and CA) do not show any base-pair mismatches with either primers or the probe. Conversely, published sequences for Kern brook lamprey (*L*. *hubbsi*, native to Merced River in California) show two mismatches in each primer, as well as the probe. There was no publically available COI sequence data for the other *Entosphenus* species to compare to this assay: Miller Lake lamprey (*E*. *minimus*, upper Klamath River basin, OR), Vancouver lamprey (*E*. *macrostomus;* Lake Cowichan on Vancouver Island, British Columbia), and Northern California brook lamprey (*E*. *folletti;* portions of the Klamath River basin in CA).

**Table 3:** List of species used for *in vitro* screening of the qPCR primers and probe. Origin refers to the waterbody for Pacific lamprey samples; origin is listed as state for all other samples.

Common name	Species name	# Samples tested	Origin
Pacific lamprey	*Entosphenus tridentatus*	5	Asotin Creek, ID
		8	Columbia River, WA/OR
		2	Imnaha River, OR
		2	Little Fall Creek, OR
		5	Lochsa River, ID
		2	Logan Creek, OR
		8	Lolo Creek, ID
		2	Middle Fork Salmon River, ID
		2	Newsome Creek, ID
		2	Selway River, ID
		2	South Fork Clearwater River, ID
		2	Tucannon River, ID
Brook trout	*Salvelinus fontinalis*	1	ID
Brown trout	*Salmo trutta*	1	OR
Bull trout	*Salvelinus confluentus*	1	ID
Chinook salmon	*Oncorhynchus tshawytscha*	1	ID
Coastal steelhead rainbow trout	*Oncorhynchus mykiss irideus*	1	MT
Columbia River redband trout	*Oncorhynchus mykiss gairdneri*	1	MT
Cutthroat trout	*Oncorhynchus clarkii*	1	WA
Dolly Varden	*Salvelinus malma*	1	AK
Mottled sculpin	*Cottus bairdii*	1	MT
Pit-Klamath brook lamprey	*Entosphenus lethophagus*	1	OR
Slimy sculpin	*Cottus cognatus*	1	MT
Sockeye salmon	*Oncorhynchus nerka*	1	ID
Pacific brook lamprey	*Lampetra pacifica*	1	Synthetically derived DNA
Unidentified brook lamprey spp.	*Lampetra* spp.	1	Hills Creek Reservoir, OR
		1	Salmon Creek, OR
		1	Upper Middle Fork Willamette River, OR
		1	Salt Creek, OR
		1	Lookout Point Reservoir, OR
		1	Little Fall Creek, OR
		1	Fall Creek, OR
		1	Dexter Reservoir, OR
		1	Echo Creek, OR

We optimized primer concentrations following methods outlined in Wilcox et al. [8] for final concentrations of 600 and 900 nM for the forward and reverse primer respectively. Using optimized assay concentrations and the cycling conditions above, we tested assay sensitivity by screening against a six-level standard curve dilution series (6 250, 1 250, 250, 50, 10, and 2 copies per 4 μl) created from target PCR product. We ran six replicates of each dilution resulting in an amplification efficiency of 97.2% (*r*^2^ = 0.979), and observed amplification in all six reactions of 10 copies per 4 μl and five of six reactions at 2 copies per 4 μl, with an average Ct = 38.4 across the five positive reactions.

Finally, we screened the assay against eDNA samples collected from eight western U.S. sites for which the presence of Pacific lamprey was known from previous electrofishing surveys (Table 4). Environmental DNA was collected from 5-l water samples following methods described in Carim et al. [9] and extracted using Qiagen DNEasy^®^ Blood and Tissue Kit following a modified protocol [10]. Using optimized assay concentrations, we analyzed these environmental samples in triplicate using the PCR recipe and cycling conditions above, but replacing 1.8 μl of water with an internal positive control consisting of water with 1.5 μl TaqMan 10 X Exo IPC Mix and 0.3 μl TaqMan 50 X IPC DNA (ThermoFisher) to test for the presence of PCR inhibitors. To test for contamination in the qPCR setup, all eDNA samples were run alongside a negative control. As expected, the assay detected Pacific lamprey eDNA in all samples collected where this species was known to be present, but not in samples collected where this species was believed to be absent. We observed no DNA amplification in qPCR negative controls and there was no evidence of PCR inhibition in any of our eDNA samples.

**Table 4:** Collection and species assemblage information for eDNA samples used to test the Pacific lamprey qPCR assay. Community assemblage information (fish species present) was obtained via a combination of eDNA analysis and personal communication with local tribal, state and federal biologists.

Waterbody (State)	Latitude	Longitude	Collection date	Fish species present*	Pacific lamprey detected? (Mean C_t_ value)
Asotin Creek (ID)	46.327268	-117.205279	8/3/2015	PALA, BLSU, BULL, CHNK, COHO, LNDC, LSSU, SCUL, SPDC, STLH	Y (37.7)
	46.331034	-117.183179	8/3/2015		Y (35.7)
	46.333719	-117.068608	8/3/2015		Y (39.1)
	46.339127	-117.056222	8/3/2015		Y (38.5)
Wenatchee River (WA)	47.487450	-120.41376	6/13/2016	BLGL, BLSU, BULL, CHMO, CHNK, COCA, COHO, LMBS, LNDC, LNSU, LPDC, LSSU, MTWF, NPKM, PEMO, PUMP, RDSS, SCUL, SMBS, SOCK, SPDC, STLH, TSSB, WALL, WSCT, YLPE	Y (35.1)
Salt Creek (OR)	43.729371	-122.421914	11/5/2015	LAMP, BRKT, BULL, CHMO, CHNK, COCT, LNDC, LSSU, MTWF, NPKM, ORCH, PEMO, RDSS, SCUL, SPDC, STLH	N (N/A)
Salmon Creek (OR)	43.746064	-122.446468	11/5/2015	LAMP, BRKT, BULL, CHMO, CHNK, COCT, LNDC, LSSU, MTWF, NPKM, ORCH, PEMO, RDSS, SCUL, SPDC, STLH	N (N/A)
Big Casino Creek (ID)	44.256347	-114.855882	8/23/2015	BRKT, BULL, CHNK, SCUL STLH, WSCT	N (N/A)
Canyon Creek (ID)	46.281419	-115.595072	10/21/2015	BRKT, BULL, LNDC, SCUL, STLH, WSCT	N (N/A)

*PALA, Pacific lamprey (*Entosphenus tridentatus*); BLGL, bluegill (*Lepomis macrochirus*); BLSU, bridgelip sucker (*Catastomus columbianus*); BRKT, brook trout (*Salvelinus fontinalis*); BULL, bull trout (*Salvelinus confluentus*); CHMO, chiselmouth (*Arcoheilus alutaceus*); CHNK, Chinook salmon (*Oncorhynchus tshawytscha*); COCA, common carp (*Carpio carpio*); COCT, cutthroat trout (*Oncorhynchus clarkii*); COHO, coho salmon (*Oncorhynchus kisutch*); LAMP, unidentified brook lamprey species (*Lampetra* spp.); LMBS, largemouth bass (*Micropterus salmoides*); LNDC, longnose dace (*Rhinichthys cataractae*); LPDC, leopard dace (*Rhinichthys falcatus*); LSSU, largescale sucker (*Catastomus macrocheilus*); LNSU, longnose sucker (*Catastomus catastomus*); MTWF, mountain whitefish (*Prosopium williamsoni*); NPKM, northern pikeminnow (*Ptychochelius oregonensis*); ORCH, Oregon chub (*Oregonichthys crameri*); PEMO, peamouth (*Mylocheilus caurinus*); PUMP, pumpkinseed (*Lepomis gibbosus*); RDSS, redside shiner (*Richardsonius balteatus*); SCUL, sculpin species (*Cottus* spp.); SMBS, smallmouth bass (*Micropterus dolomieu*); SOCK, sockeye salmon (*Oncorhynchus nerka*); SPDC, speckled dace (*Rhinichthys osculus*); STLH, steelhead trout (*Oncorhynchus mykiss*); TSSB, three-spine stickleback (*Gasterosteus aculeatus*); WALL, walleye (*Sanders vitreus*)WSCT, westslope cutthroat trout (*Oncorhynchus clarkii lewisi*); YLPE, yellow perch (*Perca flavescens*).

## Conclusions

This paper outlines the development of an eDNA assay that reliably detects Pacific lamprey in the Columbia River basin, and accurately distinguishes this species from other native lamprey (*Lampetra* spp.). As a result, this tool provides a sensitive and noninvasive sampling approach for determining the distribution of Pacific lamprey when individuals are present in low abundance, when physical sampling of individuals may be difficult or disruptive, and when accurate species level identification from morphological traits may be unreliable (but see [11] for tissue based genetic identification methods). As a result, this eDNA assay will be a valuable tool for management efforts focused on the assessment and monitoring of Pacific lamprey in the Columbia River basin. Conversely, this assay may not accurately separate other *Entosphenus* species that occur outside the Columbia River basin. As a result, this assay could serve as general assay for detection of *Entosphenus* spp. (such as *E*. *lethophagus*) where they may be sympatric. This assay may also serve to detect individual *Entosphenus* species (such as those native to the Klamath River basin) in areas where only one *Entosphenus* species is present. Nevertheless, we emphasize that this assay was developed for use in the Columbia River basin, and that validation of assay performance for other target and non-target species should be conducted prior to its use outside the Columbia River basin.
